# Effects of Salts on the Activity and Growth of “*Candidatus* Scalindua sp.”, a Marine Anammox Bacterium

**DOI:** 10.1264/jsme2.ME18068

**Published:** 2018-09-29

**Authors:** Amin Mojiri, Kazuma Nishimoto, Takanori Awata, Yoshiteru Aoi, Noriatsu Ozaki, Akiyoshi Ohashi, Tomonori Kindaichi

**Affiliations:** 1 Department of Civil and Environmental Engineering, Graduate School of Engineering, Hiroshima University 1–4–1, Kagamiyama, Higashihiroshima 739–8527 Japan; 2 Institute of Materials and Systems for Sustainability (IMaSS), Nagoya University Furo-cho, Chikusa-ku, Nagoya 464–8601 Japan; 3 Department of Molecular Biotechnology, Graduate School of Advanced Sciences of Matter, Hiroshima University 1–3–1 Kagamiyama, Higashihiroshima 739–8530 Japan

**Keywords:** “*Candidatus* Scalindua sp.”, anaerobic ammonium oxidation (anammox), salinity, ammonium consumption, inorganic carbon uptake

## Abstract

Four salts, SEALIFE (a synthetic sea salt), NaCl, Na_2_SO_4_, and NaCl+KCl, were applied to monitor the effects of salinity on “*Candidatus* Scalindua sp.”, a marine anaerobic ammonium oxidation (anammox) bacterium. The highest ammonium consumption of 10 μmol mg protein^−1^ d^−1^ was observed at 88 mmol L^−1^ of Na in the presence of NaCl. The highest inorganic carbon uptake of 0.6 μmol mg protein^−1^ d^−1^ was observed at 117 mmol L^−1^ of Na and at 16 mmol L^−1^ of K in the presence of NaCl+KCl. Thus, Na and K are both important for maintaining a high growth rate of “*Candidatus* Scalindua sp.”

Anaerobic ammonium oxidation (anammox) is a microbial process that is capable of transforming ammonium (NH_4_^+^) into dinitrogen (N_2_) gas with nitrite (NO_2_^−^) as the electron acceptor (1). Anammox bacteria mostly branch within the order *Brocadiales* in the phylum *Planctomycetes*, with five known anammox genera (16). The genus “*Candidatus* Scalindua” is primarily found in marine environments (18) and their growth rate is markedly lower (doubling time=14.4 d) than those of freshwater genera (4). To date, several “*Candidatus* Scalindua” have been successfully enriched by the supplementation of growth medium with different types of salts, such as synthetic sea salts, sea water, or NaCl (10–13, 20). We previously reported that the activity of “*Candidatus* Scalindua sp.” was significantly affected by salinity (4, 5). However, the components of salts affecting the activity and growth rate of “*Candidatus* Scalindua sp.” have not yet been identified. Salinity frequently varies in industrial wastewater rich in ammonium, such as seafood wastewater, dairy wastewater, and landfill leachate, and these variations may lead to fluctuations in salt concentrations that generate transient shock (21). Further studies to clarify the effects of salts on the activity and growth of “*Candidatus* Scalindua sp.” are needed in order to optimize the treatment of wastewater rich in ammonium and salt using “*Candidatus* Scalindua sp.”. Therefore, the aims of the present study were to (i) investigate the effects of various types of salts on the activity and growth of “*Candidatus* Scalindua sp.”, and (ii) identify optimum conditions for high ammonium consumption and inorganic carbon uptake by “*Candidatus* Scalindua sp.” under controlled salt conditions.

Anammox biomass samples were obtained from an upflow granular reactor that was scaled up from a column reactor (11, 12). The latest nitrogen loading rate (NLR), nitrogen removal rate (NRR), and total nitrogen removal efficiency were 2.9 g N L^−1^ d^−1^, 2.6 g N L^−1^ d^−1^, and 89%, respectively. The upflow granular reactor was fed a synthetic marine nutrient medium composed of 30 g L^−1^ of SEALIFE synthetic sea salt (Marine Tech, Tokyo, Japan; main components shown in Table S1), 330 mg L^−1^ (NH_4_)_2_SO_4_, 415 mg L^−1^ NaNO_2_, and other minerals previously reported (5). The dominant anammox bacterial species and community composition were identified by a phylogenetic analysis and fluorescence *in-situ* hybridization (FISH; see Supplemental Material). Batch experiments were conducted in triplicate under different salt conditions (Table 1). Biomass samples from the upflow granular reactor were homogenized and washed twice, as previously described (5). The biomass suspension (1 mL) was dispensed into 5-mL serum vials that were sealed with butyl rubber stoppers. Each vial contained 3 mL of synthetic medium, including the biomass suspension, at a final concentration of 1.2 mg protein vial^−1^, NH_4_^+^, NO_2_^−^, and KHCO_3_ (5 mM each), and salts (Table 1). The headspace was replaced with helium gas (>99.99995%) by constant vacuuming and purging. Vials were statically incubated at 28°C for 24 h to assess ammonium consumption (*i.e.*, anammox activity) and inorganic carbon uptake (related to growth rates). In the case of the inorganic carbon uptake experiment, ^14^C-labeled bicarbonate (NaH[^14^C]O_3_; specific radioactivity, 51 mCi mmol^−1^) was added at a final concentration of 10.8 μCi vial^−1^ (400 kBq vial^−1^). Analytical procedures are described in detail in the Supplemental Material and in previous studies (5, 14).

The dominant anammox species in the anammox biomass samples was identified before batch experiments. Ninety-three clones were obtained from a clone library with the *Planctomycetes*-specific primer set, Pla46f and 1390r. Seventy-eight out of 93 clones were affiliated with anammox bacteria, whereas the others were affiliated with the phyla *Chloroflexi* and *Parcubacteria*. The 78 anammox clones were grouped into one OTU (*i.e.*, OTU-A04), which was closely related to “*Candidatus* Scalindua sp.”, with 99.9% sequence identity (Fig. S1). FISH with the BS820 probe specific to “*Candidatus* Scalindua sp.” revealed that these species comprised 87% of the total bacteria (Fig. S2). These results indicate the dominance of a single anammox species in the anammox biomass samples used in the present study.

SEALIFE, a synthetic sea salt, was used to assess the effects of salinity on anammox activity in the present study because we successfully enriched a marine “*Candidatus* Scalindua” species using 35 g L^−1^ of SEALIFE (11). Ammonium consumption and inorganic carbon uptake were enhanced by increases in the concentration of SEALIFE to 2% (Fig. 1), and then decreased as SEALIFE concentrations increased from 2% to 4%. Thus, maximum ammonium consumption (9 μmol mg protein^−1^ d^−1^, Fig. 1A) and maximum inorganic carbon uptake (0.36 μmol mg protein^−1^ d^−1^, Fig. 1B) were observed at a SEALIFE salinity of 2%. These results are consistent with previous findings reported Awata *et al.* (5). Since Na is the most abundant cation in SEALIFE, as shown in Table S1, the effects of Na concentrations on ammonium consumption and inorganic carbon uptake were investigated further.

Ammonium consumption was enhanced as Na concentrations increased to 229, 88, 73, and 117 mmol L^−1^ in the presence of SEALIFE, NaCl, Na_2_SO_4_, and NaCl+KCl, respectively (Fig. 2A). Ammonium consumption was the highest (10.3 μmol mg protein^−1^ d^−1^) with 88 mmol L^−1^ Na in the presence of NaCl. It is important to note that no ammonium consumption (0.0 μmol mg protein^−1^ d^−1^) was observed at 2 mmol L^−1^ Na (*i.e.*, batch experiment 1 in Table 1). Similar to ammonium consumption, inorganic carbon uptake was enhanced by increases in Na concentrations to 229, 88, 73, and 117 mmol L^−1^ in the presence of SEALIFE, NaCl, Na_2_SO_4_, and NaCl+KCl, respectively (Fig. 2B). Inorganic carbon uptake was the highest (0.6 μmol mg protein^−1^ d^−1^) at 117 mmol L^−1^ Na in the presence of NaCl+KCl. No inorganic carbon uptake (0.0 μmol mg protein^−1^ d^−1^) was observed at 2 mmol L^−1^ Na.

In addition to the effects of Na, we investigated the effects of K concentrations on ammonium consumption and inorganic carbon uptake. Ammonium consumption was enhanced by increases in K concentrations to 11.3 and 16 mmol L^−1^ in the presence of SEALIFE and NaCl+KCl, respectively (Fig. 3A). Ammonium consumption was the highest (8.9 μmol mg protein^−1^ d^−1^) at 11.3 mmol L^−1^ K in the presence of SEALIFE, and the lowest (0.2 μmol mg protein^−1^ d^−1^) at 40 mmol L^−1^ K in the presence of NaCl+KCl. Inorganic carbon uptake was enhanced by increases in K concentrations to 11.3 and 16 mmol L^−1^ in the presence of SEALIFE and NaCl+KCl, respectively (Fig. 3B). Inorganic carbon uptake was the highest (0.6 μmol mg protein^−1^ d^−1^) at 16 mmol L^−1^ K in the presence of NaCl+KCl, and the lowest (<0.05 μmol mg protein^−1^ d^−1^) in the presence of 6 and 16.4 mmol L^−1^ of NaCl+KCl and SEALIFE, respectively.

Previous studies (2, 3, 7, 17, 22) demonstrated that high salinity reduces microbial activity and changes the microbial community structure. Kartal *et al.* (9) reported two possible outcomes of the adaptation of a biomass to salinity: (i) the acclimation of the existing population or (ii) a population shift. Commonly, the internal osmotic pressure in bacterial cells is higher than that of the surrounding environment, and, thus, pressure is exerted outward onto the cell wall; this is known as turgor pressure. When the salinity of the surrounding environment increases, cells lose water to restore the osmotic equilibrium across the cell membrane, resulting in reductions in activity and efficiency (6).

In the presence of NaCl+KCl, inorganic carbon uptake was higher than that in the presence of SEALIFE or NaCl alone (Fig. 2B and 3B). This is because SEALIFE contains a large amount of Na, but does not have a sufficiently high K content for the optimum growth and activity of “*Candidatus* Scalindua sp.”. K is the major intracellular cation in bacterial cells (8), and ranges between 0.1 and 0.6 mol L^−1^ (15). The accumulation of K as an immediate response to an osmotic upshift is observed in many bacteria (22). Stingl *et al.* (19) reported that K may exert positive effects on membrane potential adjustments and the survival of bacteria. “*Candidatus* Scalindua sp.” may consume energy for the uptake of K from the surrounding medium to maintain osmotic pressure. If sufficient K is present in the medium, “*Candidatus* Scalindua sp.” may use energy for growth, as shown in Fig. 2B and 3B. A high K concentration in the medium may increase the growth rate of “*Candidatus* Scalindua sp.”.

The key conclusions in the present study are as follows: (i) high ammonium consumption depends on the type of salt and its concentration; (ii) ammonium consumption and inorganic carbon uptake were enhanced by increases in the concentration of the synthetic sea salt SEALIFE to 2%; (iii) maximum ammonium consumption was observed at 88–173 mmol L^−1^ Na in the presence of NaCl; and (iv) inorganic carbon uptake was the highest in the presence of NaCl+KCl, indicating that not only Na, but also K is important for maintaining a high growth rate of “*Candidatus* Scalindua sp.” Based on the present results, the specific growth rates of “*Candidatus* Scalindua sp.” under different K concentrations need to be assessed for the development of nitrogen removal processes in the treatment of wastewater rich in ammonium and salt.

## Supplemental Material



## Figures and Tables

**Fig. 1:**
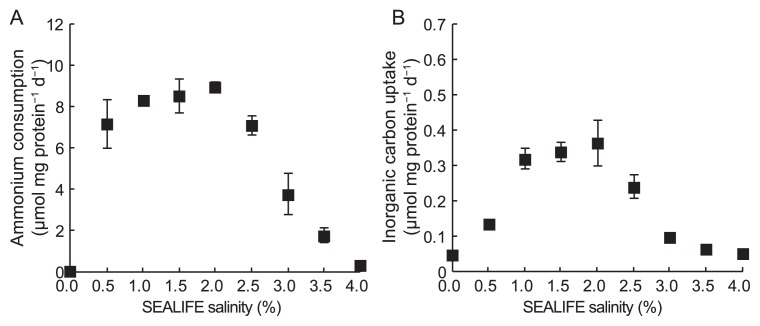
Effects of SEALIFE salinity on ammonium consumption (A) and inorganic carbon uptake (B) by “*Candidatus* Scalindua sp.”. Error bars indicate the standard deviations of triplicate batch experiments.

**Fig. 2:**
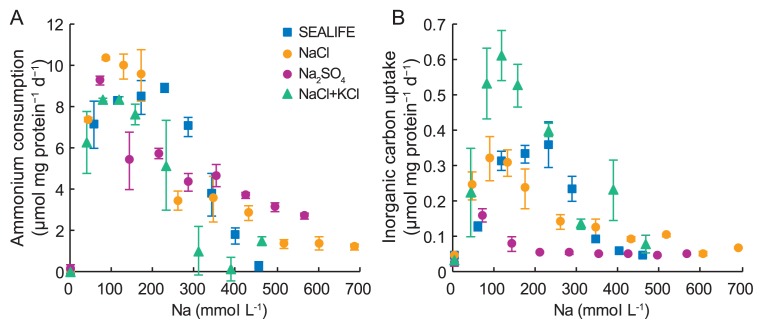
Effects of Na on ammonium consumption (A) and inorganic carbon uptake (B) by “*Candidatus* Scalindua sp.”. Error bars indicate the standard deviations of triplicate batch experiments.

**Fig. 3:**
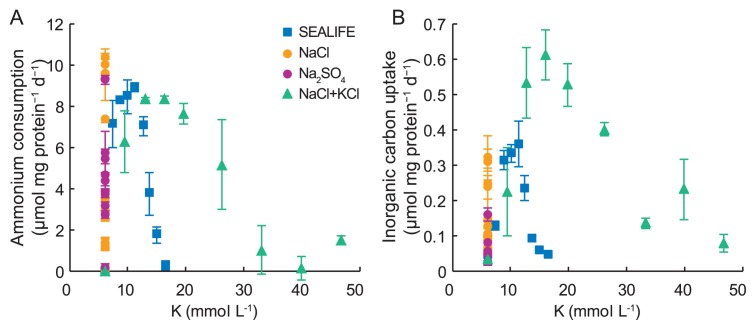
Effects of K on ammonium consumption (A) and inorganic carbon uptake (B) by “*Candidatus* Scalindua sp.”. Error bars indicate the standard deviations of triplicate batch experiments.

**Table 1:** Salt concentrations used in the present study

Batch experiment	SEALIFE			NaCl		Na_2_SO_4_		NaCl+KCl	
			
	Salinity (%)	Na (mmol)	K (mmol)	Na (mmol)	K (mmol)	Na (mmol)	K (mmol)	Na (mmol)	K (mmol)
1*	0	2	6	2	6	2	6	2	6
2	0.25	N.A.	N.A.	45	6	N.A.	N.A.	41	10
3	0.50	59	7	88	6	73	6	79	13
4	0.75	N.A.	N.A.	130	6	N.A.	N.A.	117	16
5	1.00	116	9	173	6	143	6	156	20
6	1.50	172	10	259	6	213	6	233	26
7	2.00	229	11	344	6	284	6	310	33
8	2.50	286	13	430	6	354	6	387	40
9	3.00	343	14	515	6	425	6	464	46
10	3.50	399	15	601	6	495	6	N.A.	N.A.
11	4.00	456	16	687	6	565	6	N.A.	N.A.

* Note that in batch experiment 1 (0% salinity), Na and K concentrations are non-zero because of the contribution of the synthetic medium.

N.A.; not applied.
