# Supplementary material for: Effects of Salts on the Activity and Growth of “Candidatus Scalindua sp.”, a Marine Anammox Bacterium
Source: Microbes Environ. 2018 Sep 29;33(3):336–9. doi: 10.1264/jsme2.ME18068 (PMC6167117; doi:10.1264/jsme2.ME18068)
Supplement: Supplementary file 1 [file 33_336_s1.pdf]

Supplemental Material

Effects of Salts on the Activity and Growth of "*Candidatus* Scalindua sp.", a Marine Anammox Bacterium

Amin Mojiri[1], Kazuma Nishimoto[1], Takanori Awata[2], Yoshiteru Aoi[3], Noriatsu Ozaki[1], Akiyoshi Ohashi[1], Tomonori Kindaichi[1*]

[1] *Department of Civil and Environmental Engineering, Graduate School of Engineering, Hiroshima University, 1-4-1, Kagamiyama, Higashihiroshima 739-8527, Japan*

[2] *Institute of Materials and Systems for Sustainability (IMaSS), Nagoya University, Furo-cho, Chikusa-ku, Nagoya 464-8603, Japan*

[3] *Department of Molecular Biotechnology, Graduate School of Advanced Sciences of Matter, Hiroshima University, 1-3-1 Kagamiyama, Higashihiroshima 739-8530 Japan*

* Corresponding author: Tomonori Kindaichi, E-mail: tomokin@hiroshima-u.ac.jp

Content:

Experimental procedures

Table S1 Main components of SEALIFE, used as sea salt in this study.

Fig. S1 Phylogenetic tree of the order *Brocadiales* in the phylum *Planctomycetes* based on 16S rRNA gene sequences.

Fig. S2 FISH analysis of homogenized anammox granules.

References

Experimental procedures

Phylogenetic analysis

To identify the dominant anammox species, phylogenetic analysis was performed before the batch experiments were conducted. DNA extraction, PCR amplification with the primer set Pla46f (8) and 1390r (12), cloning, and sequencing procedures have been described in a previous report (2). Sequences with ≥97% identity were grouped into operational taxonomic units (OTUs) using the neighbor-joining method with similarity corrections as implemented in the ARB software program (7). A phylogenetic tree was constructed using the maximum likelihood (RAxML) method with the general time reversible (GTR) gamma model in ARB with the SILVA release 132 SSU Ref NR 99 database (10). Rapid bootstrap analysis was performed with 1,000 runs. The sequence of the partial 16S rRNA gene was deposited in the DDBJ database under accession number LC381633.

Fluorescence in-situ hybridization (FISH)

Granule samples were homogenized and fixed in a 4% paraformaldehyde solution for 8 h at 4°C. *In-situ* hybridization was conducted according to the procedure described by Okabe *et al.* (9), and a model Axio Imager M1 epifluorescence microscope (Carl Zeiss, Oberkochen, Germany) was used for the observation. The 16S rRNA-targeted oligonucleotide probes used in this study were EUBmix, which was composed of EUB338 (1), EUB338II and EUB338III (4), EUB338IV (11), and BS820 (6). The probes were labeled with Cy3 or Alexa Fluor 488 at the 5′ end. For the quantitative determination of microbial composition in the granules, the surface fractions of the specific probe-hybridized cell area and EUBmix probe-hybridized cell area were determined after gentle homogenization (5). The average fraction was determined from 20 representative fluorescence images using ImageJ software (3).

Analytical methods

Protein concentration was measured with a Pierce BCA Assay kit (Thermo Fisher Scientific Inc., Waltham, MA, USA). Ammonium (NH_4^+) was monitored according to Nessler's method with a HACH DR-2800 instrument (Loveland, CO, USA). Ion chromatography (HPLC 20A; Shimadzu, Kyoto, Japan) using a Shodex Asahipak NH2P-50 4D anion column (Showa Denko, Tokyo, Japan) and UV-VIS detector (SPD-20A; Shimadzu) was conducted following the filtration of samples through 0.2-μm-pore membranes (Advantec, Tokyo, Japan) to monitor nitrate and nitrite.

^{14}C bicarbonate uptake was assessed by liquid scintillation counting. The biomass was collected, washed three times with phosphate-buffered saline, and mixed with the scintillation cocktail (Clear-sol I; Nacalai Tesque, Kyoto, Japan). Radioactivity was consequently determined via an LSC-5100 liquid scintillation counter (Hitachi-Aloka Medical, Tokyo, Japan). Determined radioactivity (Bq) was calculated in relation to bicarbonate concentration using the specific radioactivity (*i.e.,* 51 mCi $mmol^{-1}$).

Table S1. Main components of SEALIFE, used as sea salt in this study

Component	Concentration (mmol)*
Na^+	397
Mg^{2+}	51
K^+	9
Ca^{2+}	9
Cl^-	476
SO_4^{2-}	24

*Concentrations shown are for 35 g of SEALIFE.

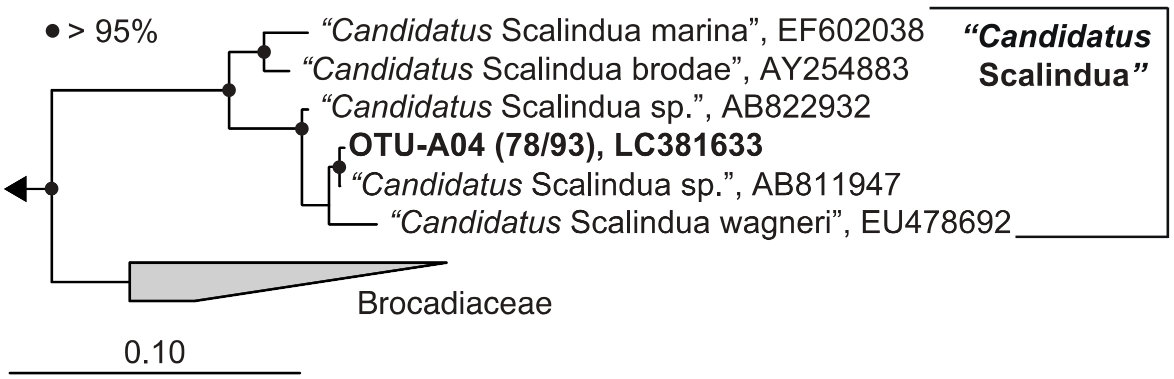

Fig. S1

Phylogenetic tree of the order *Brocadiales* in the phylum *Planctomycetes* based on 16S rRNA gene sequences. The tree was constructed with the maximum-likelihood method. The number in parentheses indicates the frequency of identical clones in the OTU. The scale bar indicates the number of nucleotide changes per sequence position. Filled circles at the nodes represent bootstrap values >95% obtained from 1,000 runs.

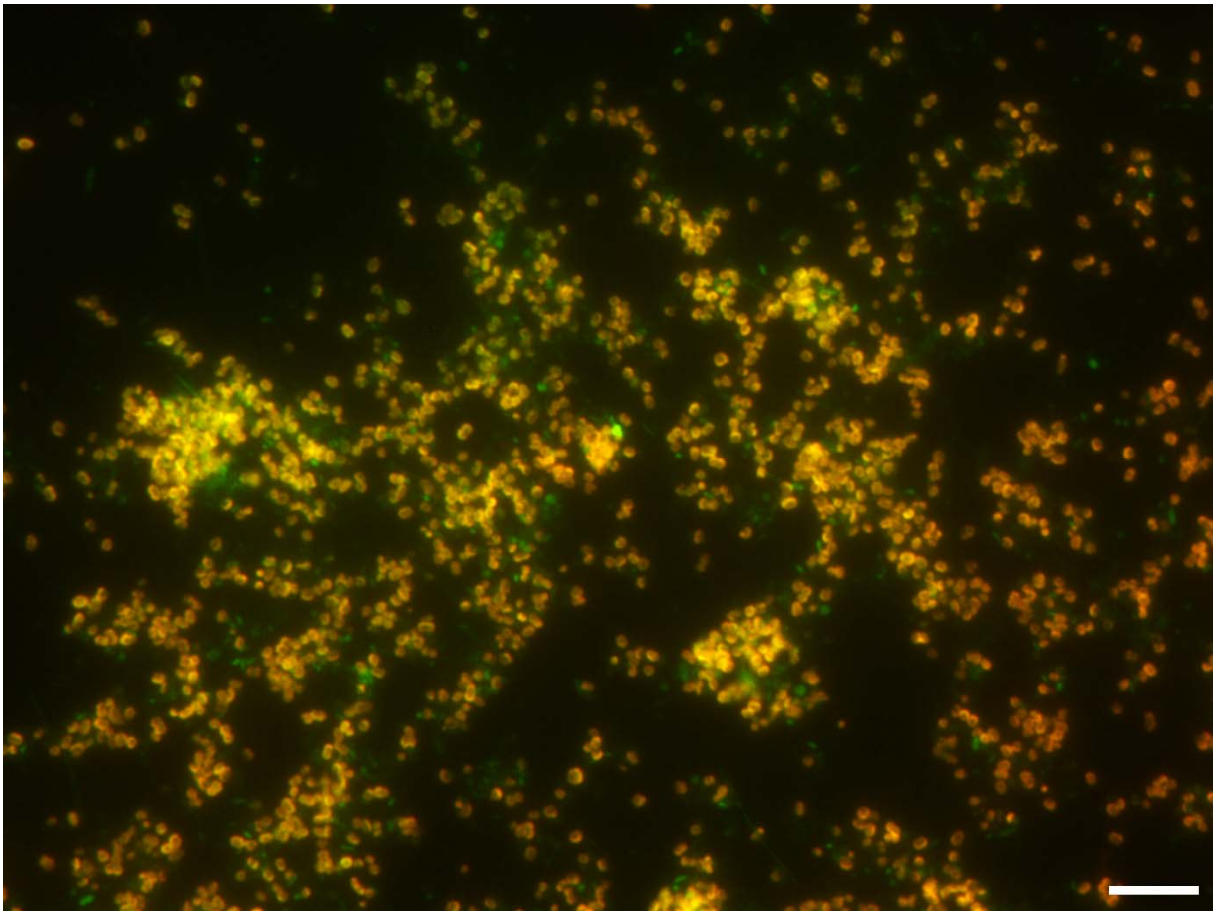

Fig. S2

FISH analysis of homogenized anammox granules. A combination of Alexa488-labeled mixed EUB338mix probes and Alexa555-labeled BS820 probe was used for *in-situ* hybridization. "*Candidatus* Scalindua" cells are yellow and non-Scalindua cells are green. Bar indicates 10 μm. Probe-defined *Candidatus* Scalindua" accounted for 87% ± 6% (means and standard deviations of 20 images).

References

1. Amann, R.I., B.J. Binder, R.J. Olson, S.W. Chisholm, R. Deverux, and D. Stahl. 1990. Combination of 16S rRNA-targeted oligonucleotide probes with flow-cytometry for analyzing mixed microbial populations. Appl. Environ. Microbiol. 56:1919-1925.
2. Awata, T., T. Kindaichi, N. Ozaki, and A. Ohashi. 2015. Biomass yield efficiency of the marine anammox bacterium, "*Candidatus* Scalindua sp.," is affected by salinity. Microbes Environ. 30:86-91.
3. Collins T. J. 2007. ImageJ for microscopy. *Biotechniques* 43, 25-30.

4. Daims, H., R. Brühl, R. Amann, K.H. Schleifer, and M. Wagner. 1999. The domain-specific probe EUB338 is insufficient for the detection of all bacteria: development and evaluation of a more comprehensive probe set. Syst. Appl. Microbiol. 22:434-444.
5. Kindaichi, T., T. Ito, H. Satoh, and S. Okabe. 2004. Ecophysiology interaction between nitrifying bacteria and heterotrophic bacteria in autotrophic nitrifying biofilms as determined by microautoradiography-fluorescence in situ hybridization. Appl. Environ. Microbiol. 70:1641-1650.
6. Kuypers, M.M.M., A.O. Sliekers, G. Lavik, M. Schmid, B.B. Jørgensen, J.G. Kuenen, J.S.S. Damsté, M. Strous, and M.S.M. Jetten. 2003. Anaerobic ammonium oxidation by Anammox bacteria in the Black Sea. Nature 422:608-611.
7. Ludwig, W., O. Strunk, R. Westram, *et al.* 2004. ARB: A software environment for sequence data. Nucleic Acids Res. 32:1363-1371.
8. Neef, A., R. Amann, H. Schlesner, and K.H. Schleifer. 1998. Monitoring a widespread bacterial group: in situ detection of planctomycetes with 16S rRNA-targeted probes. Microbiology 144:3257-3266.
9. Okabe, S., H. Satoh, and Y. Watanabe. 1999. In situ analysis of nitrifying biofilms as determined by in situ hybridization and the use of microelectrodes. Appl. Environ. Microbiol. 65:3182-3191.
10. Pruesse, E., C. Quast, K. Knittel, B. Fuchs, W. Ludwig, J. Peplies, and F.O. Glöckner. 2007. SILVA: a comprehensive online resource for quality checked and aligned ribosomal RNA sequence data compatible with ARB. Nucleic Acids Res. 35:7188-7196.
11. Schmid, M.C., B. Mass, A. Dapena, *et al.* 2005. Biomarkers for in situ detection of anaerobic ammonium-oxidizing (anammox) bacteria. Appl. Environ. Microbiol. 71:1677-1684.
12. Zheng, D., E.W. Alm, D.A. Stahl, and L. Raskin. 1996. Characterization of universal small-subunit rRNA hybridization probes for quantitative molecular microbial ecology studies. Appl. Environ. Microbiol. 62:4504-4513.
